# A “Shallow Phylogeny” of Shallow Barnacles (*Chthamalus*)

**DOI:** 10.1371/journal.pone.0005567

**Published:** 2009-05-15

**Authors:** John P. Wares, M. Sabrina Pankey, Fabio Pitombo, Liza Gómez Daglio, Yair Achituv

**Affiliations:** 1 Department of Genetics, University of Georgia, Athens, Georgia, United States of America; 2 Department of Ecology, Evolution, and Marine Biology, University of California Santa Barbara, Santa Barbara, California, United States of America; 3 Departamento de Biologia Marinha, Universidade Federal Fluminense, Niterói, Rio de Janeiro, Brazil; 4 School of Natural Science, University of California Merced, Merced, California, United States of America; 5 The Mina and Everard Goodman Faculty of Life Sciences, Bar Ilan University, Ramat Gan, Israel; University of North Carolina at Chapel Hill, United States of America

## Abstract

**Background:**

We present a multi-locus phylogenetic analysis of the shallow water (high intertidal) barnacle genus *Chthamalus*, focusing on member species in the western hemisphere. Understanding the phylogeny of this group improves interpretation of classical ecological work on competition, distributional changes associated with climate change, and the morphological evolution of complex cirripede phenotypes.

**Methodology and Findings:**

We use traditional and Bayesian phylogenetic and ‘deep coalescent’ approaches to identify a phylogeny that supports the monophyly of the mostly American ‘fissus group’ of *Chthamalus*, but that also supports a need for taxonomic revision of *Chthamalus* and *Microeuraphia*. Two deep phylogeographic breaks were also found within the range of two tropical American taxa (*C. angustitergum* and *C. southwardorum*) as well.

**Conclusions:**

Our data, which include two novel gene regions for phylogenetic analysis of cirripedes, suggest that much more evaluation of the morphological evolutionary history and taxonomy of Chthamalid barnacles is necessary. These data and associated analyses also indicate that the radiation of species in the late Pliocene and Pleistocene was very rapid, and may provide new insights toward speciation via transient allopatry or ecological barriers.

## Introduction

The high intertidal barnacles of the genus *Chthamalus* have a high profile of systematic and biogeographic exploration, in part due to the confusing degree of morphological plasticity and similarity among species – a trait that promoted Darwin's [1] interest in the taxonomy and evolution of barnacles. Additionally, the genus *Chthamalus* has proven to be an excellent group of organisms for experimental ecology, in particular pertaining to interspecific competition [2], [3], [4]. As this genus tends to be found in the highest reaches of the intertidal zone, it experiences some of the most dramatic daily and seasonal physiological transitions, and is one of several taxa that can be used as a clear indicator that global climate change is forcing the redistribution of many marine species [5], [6].

The genus is globally distributed, locally abundant, and relatively diverse, with over 20 nominal species [7], [8]. The distributions of these species, and heritable diversity within each, are largely determined through abiotic mechanisms because the sole mechanism of dispersal is the planktotrophic larval stage, which may require 4–6 weeks of development before an individual is competent to settle. Thus, changes in the nearshore environment and changes in nearshore ocean circulation are likely to have significant effects on the demographic isolation of populations and the diversity each population harbors. Hybridization between species is thought to be rare [9]; however, the morphological and ecological similarity among many of these species, with broad phenotypic variation within species and a large capacity for larval dispersal, has made distinguishing species for biogeographic or evolutionary analysis difficult. Understanding the impact of cryptic species on biogeographic patterns is critical for evaluation of what generates and maintains diversity [10].

Among the characters that are often used in diagnosing species in this family and genus, many are of questionable value [11]. Pilsbry [12] initially separated the genus into two groups based on mandibular shape; Nilsson-Cantell [13] subsequently named these groups the quadridentoid Stellatus group and the tridentoid Hembeli group. Zullo [14] determined that the tridentoid form was the plesiomorphic state – and the closely related tropical genus *Euraphia* (now largely *Microeuraphia*, under the Euraphiinae[11]) bears this trait – while the quadridentate genera (*e.g.*, *Chthamalus* under Chthamalinae) were considered to bear the more derived trait. However, Pope[15] argued that “the dividing line between those species with the so-called tridentate or *hembeli* jaw and those with the quadridentate or *stellatus* kind of mandible is sometimes a rather hazy one.” It is also clear that characters found on feeding appendages (cirri) that are often used to discriminate subgroups of the genus *Chthamalus* (e.g., Dando and Southward 1980 [16]) are quite variable within species and may themselves be problematic characters for discriminating the effects of phylogenetic history and current ecology [15], [16], [17].

With such variation in these key traits, they are nevertheless often used for taxonomic purposes [18]. Species-level taxonomy has gone through a range of reductions [19] and expansions [9], [16] as more data become available. Molecular data in particular have been invaluable for characterizing these taxa, in terms of identifying new species or lineages (e.g., [7], [20]) and phylogenetic exploration of the utility of known morphological traits for identification [21], [22].

Wares [21] attempted to clarify the relationships of the Tropical Eastern Pacific (TEP) species complex; although the ‘fissus group’ *sensu* Dando and Southward [16] appeared to be monophyletic, there were inadequate data to address larger phylogenetic questions, and some confusion remained about the phylogeny of the genus overall [7]. Although molecular approaches have been quite useful for delimiting cryptic species in this group, it is difficult to find sufficient multilocus variation to identify the pattern of divergence at deeper nodes; most nuclear loci are not sufficiently variable to evaluate Miocene divergences [23]. Because most phylogenetic studies have been regionally restricted [21], [24], we are still deficient in complete understanding of the phylogeny of this important intertidal taxon. Knowing the pattern and timing of radiation in this group may help illuminate the mechanisms involved in species divergence.

Here, we examine a broader collection of species within *Chthamalus*, including species from all four subgeneric groupings defined by Dando and Southward [16] based on the internal characters discussed above. We include closely related species in the genus *Microeuraphia* from the TEP and Caribbean to generate at least two pairs of geminate (across the Panamanian Isthmus) taxa for calibration of the timing of species radiation. Data from these species are used to address whether the morphological characters noted above are useful and consistent predictors of phylogeny, and what global or regional events may be temporally associated with the radiation of species along the North and Central American coasts. Most importantly, we address these questions using a variety of phylogenetic approaches, including consideration of the ‘deep coalescent’ [25] methods that account for intraspecific variation and shared allelic variation among species to describe more precisely the radiation of species and to define portions of the phylogeny that are likely to be problematic because of rapid ancestral radiations. These methods, applied to a species group thought to have radiated in the recent Miocene [26], provide greater flexibility in the design of future studies to address the radiation in more detail.

## Results

### Verification and Preliminary Phylogenetic Analysis

At the mitochondrial **16S** locus, we obtained 499 bp of aligned sequence data for 252 individual barnacle specimens (Genbank FJ862065-306). Of these characters, 302 were constant and 24 were uninformative; 173 characters are parsimony informative. Saturation tests indicated strong phylogenetic signal at this locus, as did a plot of transitions and transversions against HKY-corrected genetic distances. Both types of substitutions showed effectively linear increasing functions with genetic distance. MP bootstrap analysis of the mt16S partition indicated a number of interior nodes with <60% bootstrap support (similar result using NJ), with a mean bootstrap value across all internal nodes of 77.4% (73.2% for NJ). All individuals identified to species with mtCOI were consistently delineated the same way with mt16S with greater than 95% bootstrap support for each taxon, with the exceptions of the two clades of *C. southwardorum* that were identified with mtCOI, and *C. hedgecocki* and *C. panamensis* (but which combined have bootstrap support >95% under each analysis), suggesting less phylogenetic support for those taxonomic and/or phylogeographic patterns. No individuals or data partitions were excluded from subsequent analysis based on this gene alone. The best-fit model of molecular evolution at this locus was TVM with invariant sites (p = 0.44) and gamma-distributed site rates (α = 0.76). Base frequencies were heavily A/T biased (70%); the chosen model was used for subsequent corrected distance and likelihood analysis, with a 6-parameter invariant/gamma-distributed rate set for MrBayes.

At the nuclear **LTRS** locus, we obtained 434 bp of aligned sequence data for 163 specimens (Genbank FJ862503-665). Of these characters, 224 were constant and 7 were uninformative; 203 characters were parsimony informative. Saturation tests indicated poor phylogenetic signal at this locus, and transitions appeared to be heavily saturated on a plot of substitution type against HKY-corrected genetic distances. It should be noted that although mtCOI has already proven to be a poor locus for phylogenetic reconstruction for this genus, it has better indicators under both of these tests than the LTRS locus did. Heuristic MP search of complete data, as well as transition-only and transversion-only partitions, generated a pattern suggesting extreme homoplasy among individuals of the outgroup taxon *Notochthamalus scabrosus*, with individuals scattered throughout basal nodes and branches of the tree. These individuals were removed from subsequent analysis at this locus, as the data may be derived from a paralogous gene region or other artifact. Extreme conflicts (e.g., multiple distant clades of the same nominal species) were also identified for *C. malayensis* and *C. neglectus*, for which there were few sequences to begin with, so these taxa were also excluded. Finally, n = 8 individuals from the TEP (primarily *C. southwardorum* and *C. panamensis*) deviated strongly in phylogenetic placement from nominal conspecifics. Because in general the TEP species tended to each be reciprocally monophyletic with similar topology as in other gene regions, and at least 10 individuals from each of these taxa remained after the 8 sequences are removed, we excluded the deviant sequences from further analysis at this locus. From the subtracted data set, the average internal node bootstrap support was 59.7%, with individual species and internal node support substantially lower using transversions only (similar results using NJ). The best-fit model at this locus was HKY with gamma-distributed site rates (α = 0.39).

At the nuclear **NAKAS** gene, we obtained 274 bp of aligned sequence data for 109 individual barnacle specimens (Genbank FJ862666-774). Of these characters, 185 were constant and 12 were uninformative; 77 characters were parsimony informative. Saturation tests indicated little-to-moderate saturation at this locus, and a plot of transitions and transversions against corrected genetic distances suggested that this locus behaves well for phylogenetic analysis. Bootstrap support for the unweighted MP tree suggests expected species-group support (e.g., *C. dalli* and *C. challengeri* grouped with 97% bootstrap support) but very little resolution at deeper nodes of the tree (similar results with NJ). The best-fit model of molecular evolution at this locus was F81 (substitution rates equal) with invariant sites (p = 0.47) and gamma-distributed rates (α = 0.65). Base frequencies were G/C biased (62%); both models were compared for independent analyses at this locus.

At the nuclear **EF1a** locus, we obtained 464 bp of aligned sequence data for 196 individual specimens (Genbank FJ862307-502). Of these data, 319 characters were constant and 119 were parsimony informative, with no gaps. Saturation tests indicated little saturation at this locus, and a plot of substitution types against HKY-corrected distances suggested this locus could behave well for phylogenetic anlaysis. Most species delineated by mtCOI were well supported by EF1a; *C. dalli* was only separable from *C. challengeri* in 57% of bootstrap replicates, and the cryptic group of *C. angustitergum* was separable 59% of the time. The TEP group was again poorly separated. Deeper nodes were not well supported in the MP bootstrap analysis, with average internal node bootstrap support only 52%; results were comparable for NJ bootstrap. The best-fit model of molecular evolution at this locus was TrN with invariant sites (p = 0.52) and gamma rates (α = 0.97).

We obtained 817 bp of aligned sequence data at the nuclear **18S** rDNA locus for 20 specimens across 20 species. Of these data, only 9 characters were parsimony informative. Saturation testing indicated little saturation; however, this locus harbored too little variation to reliably distinguish even the outgroup taxa from our ingroup (although the *Microeuraphia* species grouped together with 84% support, no other species pair had higher than 67% support and the tree remained otherwise completely unresolved). Since there was so little variation among even distantly related taxa at this locus, it was excluded from subsequent analysis.

For the concatenated data set, we obtained a complete data set of 1671 characters for 145 taxa. Of these data, 558 characters were parsimony-informative. Bootstrap analysis was consistent with single-gene approaches in the strong support for individual taxa. Excluding the bootstrap support for these tip clades, the average bootstrap support for internal nodes was 79%, ranging from ≤50 to 100%. Similar results were obtained using NJ and Bayesian analysis.

Following these preliminary analyses, two cryptic clades of apparent *Microeuraphia* species, both sampled in the TEP, were excluded because of inconsistencies among gene partitions and inconsistent placement among topologies chosen within gene partitions and concatenated data sets. One of these clades was related to the Jalisco specimens of Wares [21] and no morphological specimens remained; the other clade was represented by only 3 individuals that suggested a reciprocally monophyletic clade at mtCOI, but placement of these individuals varied widely among other genes. Final analysis proceeded with the reduced data set for full Bayesian analysis, with support under other criteria (parsimony, distance) indicated where appropriate.

### Full Phylogenetic Analysis of Chthamalus

**Figure 1:**
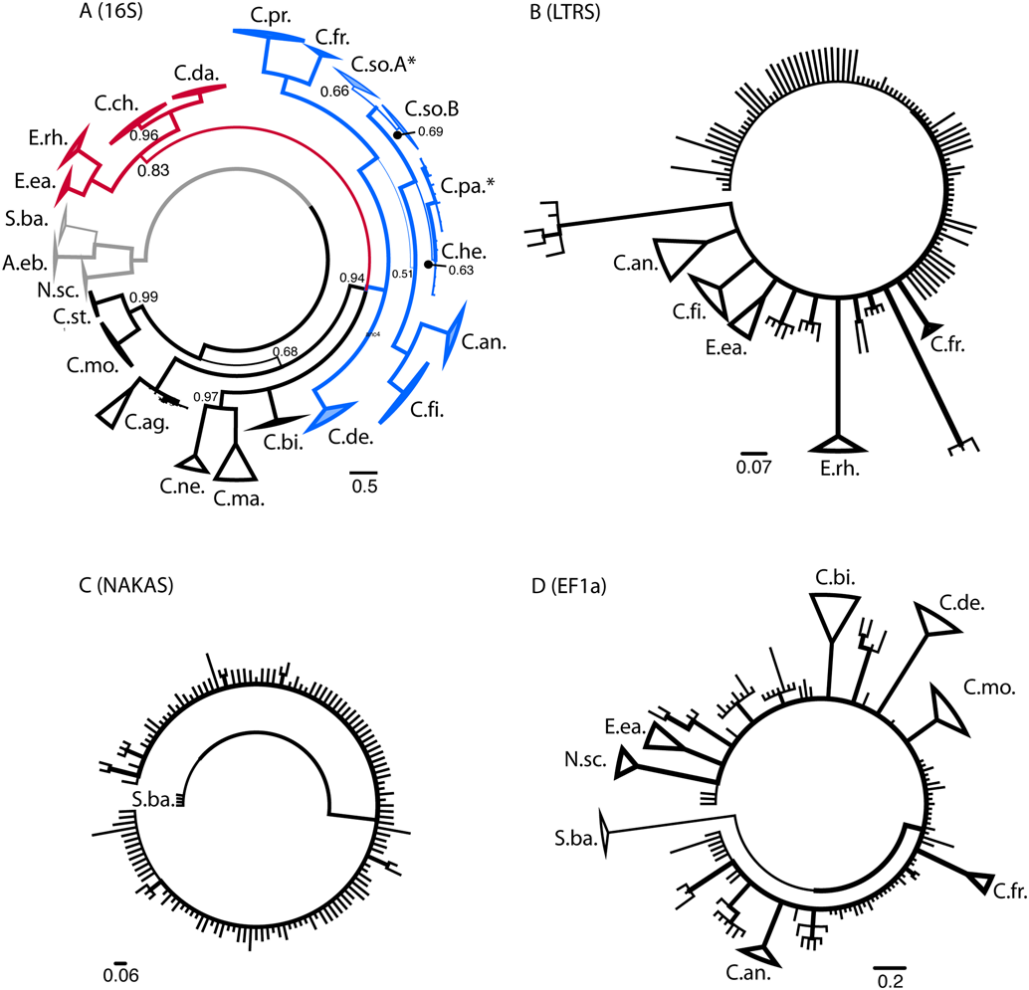
Consensus gene trees from individual loci. Phylograms generated from individual Bayesian analysis of (A) mt16S, (B) nLTRS, (C) nNAKAS, and (D) nEF1 gene regions. Nodes with less than 0.50 posterior probability collapsed. Illustrated as “circle trees” to provide spatial separation at small scale (and for cirripedic appearance). Posterior support for nodes illustrated via thickness of branches; posterior probabilities of nodes shown in (A) mt16S except where posterior  = 1.0, same thickness scale used on trees (B–D). Taxa that were found to be wholly monophyletic (based on categorization with mtCOI) are indicated as cartoons with depth of intraspecific variation portrayed as depth of triangle (using FigTree v.2.1). At (A) mt16S, all species are indicated, with asterisks indicating TEP species for which a single individual appeared in the incorrect but otherwise monophyletic grouping of that taxon. Species abbreviated with first letter of genus and first two letters of specific epithet, except for *C. angustitergum* = *C. ag*. As with parsimony and neighbor-joining analysis, there is little deep-node resolution at the nuclear gene regions. In (A) mt16S, outgroup taxa are indicated in grey; ‘challengeri group’+*Microeuraphia* indicated in red (posterior probability 0.83); ‘fissus group’ indicated in blue (posterior probability 1.0).

Given the final data set, re-analysis of each gene proceeded individually and with the concatenated complete data. Results for individual genes are shown in Figure 1. As suggested by analysis of the unfiltered data, the nuclear genes generated less consistent support for some individual taxa and most of the deeper nodes of the phylogeny. Figure 1A shows an almost-fully resolved phylogeny at mt16S, with a mean posterior probability at internal nodes of 0.879. The ‘*fissus* group’ was monophyletic and completely supported, as was the ‘*challengeri* group+*Microeuraphia*’ clade; however, the tree was not resolved for the relationship of these two clades, *C. bisinuatus*, and the ‘*malayensis* group’. The three nuclear genes (Figure 1B–D) provided strong support for some species-level differentiation, but internal nodes were lacking in support. This was consistent with low mean internal node bootstrap support at these loci (e.g., 59.7% at nLTRS as shown above). The nLTRS locus exhibited a homoplasy index in parsimony analysis of 0.40, with a rescaled consistency index of only 0.56. Similar results were obtained at all 3 nuclear loci.

Concatenated analysis of sequence data generated a more reliable tree. Including all 4 gene regions allowed reconstruction of sister taxon relationships with high confidence (e.g., *C. stellatus*/*C. montagui*, *C. challengeri*/*C. dalli*, *C. angustitergum*/*C. bisinuatus*, *C. fissus/C. anisopoma*, *C. stellatus/C. fragilis* all supported with 100% posterior probability), but the backbone of the tree had almost no statistical support. The ‘*fissus* group’ was poorly resolved and had only 0.50 posterior probability as a clade, for example, and overall the internal node mean posterior support was only 0.73 (including the branches supporting sister taxa; without these branches mean posterior support was only 0.60). Subsequent analysis excluded each of the four primary loci individually to generate a concatenated 3-locus phylogeny; significant improvement was obtained through exclusion of the nLTRS locus. This approach was confirmed through pairwise partition homogeneity tests of our loci; the nLTRS locus, in all pairwise comparisons, appeared to be significantly incompatible (p<0.001). Only 1 of 3 remaining pairwise tests (mt16S vs. nEF1a) generated a similar result, but each of these loci were easily compatible with nNAKAS (p>0.025 for both). Nearly identical results were obtained when invariant characters were excluded, as shown in simulation studies [27]; since significance probabilities for this test tend to indicate improved phylogenetic signal when p>0.01, and the test in general is overly conservative [28], [29], nEF1a was retained.

**Figure 2:**
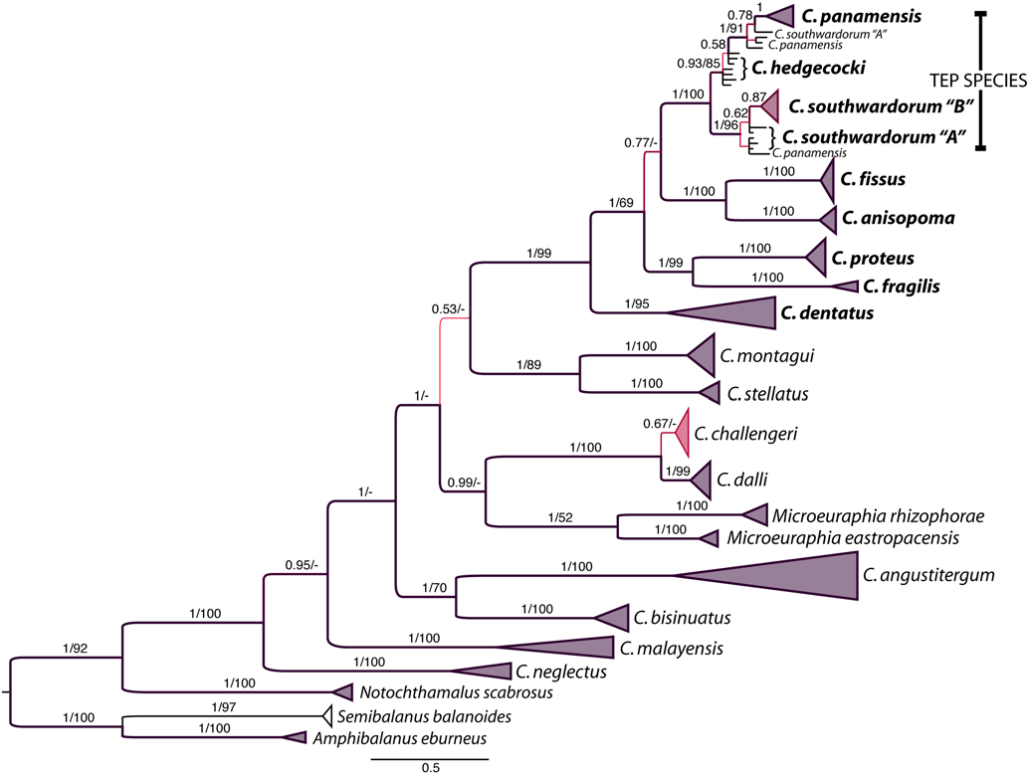
Concatenated analysis of mt16S, nNAKAS, and nEF1. Phylogram generated from Bayesian analysis of concatenated mt16S, nNAKAS, and nEF1 gene regions with unlinked substitutional models. For each clade, the posterior probability is indicated (with numerical support and correlated branch thickness/color), followed by the unweighted parsimony nonparametric bootstrap support at the same node (separated by ‘/’). Correlation of Bayesian and parsimony support indices was only 0.44 at internal nodes, with similar support from neighbor-joining bootstrap analysis. The “fissus group”, indicated with species names in bold, is monophyletic and strongly supported under all 3 methods (posterior probability 1.0, MP bootstrap 99%, NJ bootstrap 90%).

Figure 2 illustrates this ‘final’ phylogenetic analysis, which provides evidence for a wholly monophyletic and strongly supported ‘*fissus* group’, and high statistical support throughout the tree with the exception of a statistical polytomy among the ‘*challengeri* group+*Microeuraphia*’, ‘*C. stellatus*+*C. montagui*’, and ‘*fissus* group’ clades. These results were reflected by poorly correlated (r = 0.32, p>0.15) parsimony bootstrap support; many deep nodes have poor support under parsimony that were well reconstructed by the independent-model likelihood approach.

### Bayesian Estimate of Species Tree

While this analytical approach was used to attempt resolution of the most difficult nodes on our reconstructed phylogeny through using intraspecific variation as a cue to temporal order of species divergence, we were unable to reconstruct a satisfactory species tree after repeated attempts at parameter optimization. In every individual mb-best analysis, the duplicate runs did not converge, with average standard deviation of split frequencies larger than 0.10. This result was obtained whether using the whole concatenated data set or excluding LTRS as above. Final analytical results approximated the species tree shown in Figure 2, but with very low differentiation among possible topologies in terms of their posterior probability. No single species tree topology carried a posterior probability >0.016, with the top 19 topologies (out of 2963 recovered) accounting for the first 10% of the cumulative posterior distribution. The subgroup clades indicated in Figure 2 were not wholly monophyletic with respect to prior taxonomy, *e.g. C. dentatus* fell out with the basal taxa *C. neglectus* and *C. malayensis* in some of these ‘high probability’ trees, although the general relationships in Figure 2 that are of note here – the monophyly of the ‘*fissus* group’ (other than *C. dentatus*, in this case) and the recent relationship between species in the ‘*challengeri* group’ and *Microeuraphia* – were supported in the final consensus tree and the most probable ultimate topologies.

### Species Radiation Timing

The divergence of tropical barnacles with the rise of the Panamanian Isthmus not only generates diversity at the species level, it also allows calibration of the rate of molecular evolution for novel taxa and, in this case, novel genes. Table 1 illustrates the distribution of rates associated with comparisons of the two geminate species of *Microeuraphia* and the mean divergence (net nucleotide distance) among trans-Isthmian *Chthamalus* species (*i.e. C. proteus+C. fragilis vs. C. fissus+C. anisopoma*+the TEP species). Phylogenetic reconstruction at each gene region deviated significantly from the expectations of a molecular clock (evaluated by standard likelihood ratio test). Additional corrections to this model involve additional assumptions and may still be misleading [21], and no precise temporal hypotheses are being evaluated with these data, therefore no additional testing or adjustment of rates was done. Overall, the observed rate at mt16S (about 1.25% per million years) was comparable to previous studies at this locus, while the first-time rate estimates at the other three loci appeared to be about half as fast (Table 1). At the nLTRS locus, substantial differentiation between *M. rhizophorae* and *M. eastropacensis* (see Figure 1B) suggested a much higher rate than estimation based on divergence in *Chthamalus* alone. Given the unusual patterns of substitution noted in other taxa (*e.g. N. scabrosus*) this gene region may violate assumptions of rate constancy.

**Table 1:** Calibrated rates of molecular evolution per locus.

Locus	Accept clock by LRT?	d_A_ *Microeuraphia*	d_A_ *Chthamalus* (mean)	Rate distribution	%/m.y.
**16S**	no	0.059±0.011	0.106±0.019	2.59±1.17×10^−8^	1.29±0.58
**EF1**	no	0.041±0.011	0.037±0.009	1.23±0.49×10^−8^	0.62±0.24
**NAKAS**	no	0.078±0.269	0.031±0.029	1.26±1.62×10^−8^	0.63±0.81
LTRS*	no	*0.182*±*0.037*	0.046±0.011	1.45±0.65×10^−8^	0.73±0.33

None of the individual data sets carry an appropriate fit to the standard molecular clock model (rejected at p<0.001). Net nucleotide distance is calculated for both trans-Isthmian taxa as appropriate; for *Chthamalus*, the mean distance for comparisons among all Pacific ‘*fissus* group’ species with both *C. proteus* and *C. fragilis* was calculated with standard deviations. Rate distributions (and calculations per million years) are estimated using a range of dates 3.0–3.5 mya for the closing of the Panamanian Isthmus. Rate distributions for nLTRS exclude the *Microeuraphia* comparison as all *Chthamalus* comparisons were extremely similar with low variance.

Knowing that several speciation events have occurred in *Chthamalus* after the closure of the Panamanian Isthmus (*C. proteus−C. fragilis*, and the divergence between the TEP species and the *C. fissus−C. anisopoma* pair, and divergences within each of those clades) suggests high levels of speciation in the Pleistocene. A character-independent analysis using the BiSSE model was not able, however, to distinguish the gene tree for *Chthamalus* from one in which speciation rate is equivalent to extinction rate (i.e. a Yule uniform speciation model, results not shown). Coding the species in the ‘fissus group’ separately from other species in the phylogeny (to examine the diversification of this group in the Americas using the character-associated diversification BiSSE analysis) identified slightly elevated speciation rate λ for this group (4.104 versus 3.859), but the extinction rate μ was considerably lower (7.826×10^−6^ versus 2.524). However, a complete phylogeny of the remaining groups will be necessary to fully evaluate this pattern.

## Discussion

Two consistent results are gained from this extensive phylogenetic analysis of Chthamalid barnacles: first, a dominant upper intertidal group in North America and northern South America, the ‘*fissus* group’ sensu Dando and Southward [16], is monophyletic. The internal morphological characters (bidenticulate setae with basal guards on cirrus II, absence of conical spines on cirrus I) are not exclusive to this group, and confirmation with molecular data is important for understanding the radiation of this genus along both coasts of the Americas. Pitombo (in prep) has identified a novel synapomorphy for this subgeneric group, and the monophyly of all four groups (based on a combination of presence/absence of the characters above) still needs to be evaluated (see below).

The second result, and one that is of greater phylogenetic interest, is that *Microeuraphia* appears not to be the ancestral or sister taxon to *Chthamalus* as is frequently suggested in the literature [15], [18], but is consistently nested within *Chthamalus* and is closely affiliated with species in the current ‘*challengeri* group’. These results are supported by an independent analysis (Y. Achituv, unpublished) and are consistent across various phylogenetic models and reconstruction criteria. Here we use three outgroup genera, two (*Semibalanus* and *Amphibalanus*) from families Archaeobalanidae [18]and Balanidae (Leach 1817) and all other species in this study from the family Chthamalidae (Darwin 1854), and obtain strong bootstrap and posterior probability for the inclusion of *Microeuraphia* – originally presumed to be a fourth outgroup taxon – within the *Chthamalus* clade. Given the uncertainty at some internal nodes on this tree, we additionally examined the likelihood that *Chthamalus* is monophyletic with all other genera forming a reciprocal clade by performing a Kishino-Hasegawa test to compare unconstrained trees vs. trees constrained to include *Semibalanus*, *Amphibalanus*, *Notochthamalus*, and *Microeuraphia* in the outgroup. This test indicated a significant (p<0.001) difference between these trees, with a mean difference in log *L* of 62.54, supporting the monophyly of *Chthamalus* (including *Microeuraphia*). Of course, only two species of *Microeuraphia* are included in the present study, but two other cryptic lineages present in preliminary analyses (identified at the level of mtCOI, but with inconsistent results in other loci) indicated the same pattern, and other studies (Y. Achituv, unpublished) also show *Microeuraphia* nested within *Chthamalus*. We also have specimens of *M. intertexta* and *M. aestuarii* but did not obtain sufficient data from these species to include; further work can confirm this pattern, but we see no reason to doubt this placement and hence recommend taxonomic revision. Our results reflect some uncertainties already indicated about the relationships of the subfamilies and genera of the Chthamalidae [11].

Wares[21] had used *Microeuraphia* as the sole outgroup taxon for rooting a mitochondrial phylogeny of *Chthamalus*. This may have led to a variety of inconsistencies that are resolved in this study by more extensive genomic and species sampling as well as more detailed curation of morphological specimens. The phylogeny shown in Figure 2 is effectively only a phylogeny of the consistent, with regard to criterion (the same topology obtained using NJ, MP, and Bayesian/likelihood approaches), gene region, and OTU. Specimens that generated more complex patterns – including those noted in results such as the LTRS data in *Notochthamalus* and some TEP specimens that were found in different clades depending on the gene region analyzed – are not presented in the final analysis. There is likely more phylogenetic and biological complexity to be represented due to potential hybridization among taxa and other forms of intraspecific variation that are not yet accounted for. Since some of these gene regions are novel for phylogenetic analysis, we may also need to learn more about the mutational models that generate both high diversity and high homoplasy, as in the nLTRS gene region. The concatenated data set including the nLTRS locus had significantly poorer statistical resolution than the analysis when this locus is excluded, despite the large number of informative characters. High levels of substitutional saturation are likely the problem, suggesting that even in this intron region there may be areas of higher mutational frequency.

Generating genetic data that are sufficiently variable for phylogenetic analysis of species that appear to have largely radiated in the Miocene is challenging. As such, we recovered loci that not only differentiate the species well (though not always with high statistical support, Figure 1) but also harbor intraspecific variation. These data should have been appropriate for Bayesian estimation of the species tree [30], incorporating the concept of the ‘deep coalescent’ [25] in the reconstruction of relationships among shallow-water barnacles. The probability of allelic diversity in ancestral populations – such as the Miocene progenitor population to the *challengeri*, *stellatus*, and *fissus* groups (and *Microeuraphia*) – sorting locus by locus in a way that does not necessarily follow the containing species tree is by now a well known problem [31]. Other attempts to use this new method for estimating the species tree from multi-allelic information within each taxon have provided results more consistent with expected and observed phylogeny using more traditional methods [32], though the mb-best approach often indicated much weaker node support across the phylogeny as with our results. Their analysis was primarily focused on intraspecific variation and could apply to the TEP taxa; in all mb-best analyses we did recover a monophyletic TEP clade within the ‘*fissus* group’, for example. Given the exceedingly low posterior probabilities for most nodes found in our mb-best analysis (as well as in [32]), along with topologies that largely disagree with traditional phylogenetic approaches (Figure 2 and methods above) as well as traditional taxonomy, we assume that the radiation of these species was simply very rapid at certain points in their history (i.e. associated with nodes around the noted region in Figure 2), leading to extensive hemiplasy [31], and that other approaches for inferring the importance of deep coalescent events [33] in our data may prove useful.

The rapid radiation of species certainly applies to the morphologically and ecologically cryptic TEP species (*C. southwardorum*, *C. hedgecocki*, and *C. panamensis*). Given their position in the topology and our understanding of trans-Isthmian relationships, high level of speciation must have taken place in the Pleistocene in the tropical eastern Pacific. It may be questioned how real these species are under the conditions of DNA barcoding: while there are 4 reciprocally monophyletic taxa under analysis of mtCOI (Appendix I), in no case do these groups fit the “10× rule” [34] that might indicate substantial temporal and demographic isolation that may be predictive of reproductive isolation. However, the taxa are also recovered using 16S as distinguishable clades, though not reciprocally monophyletic (i.e., *C. southwardorum* B, the northern clade, appears to be nested within the southern group of *C. southwardorum* A). They are statistically distinguishable but not monophyletic groups at the nuclear loci (Meyers, Pankey, and Wares in prep). While there are clear phylogeographic patterns resolved in *C. southwardorum* (and also *C. angustitergum* on the Caribbean coast), it is important to recognize that these patterns may require substantial effort to resolve further. Dando and Southward [16] illustrate the potential for large disjunction in available habitat for *C. angustitergum* on the Central American coast suggesting that while the divergence between populations of this species in Panama and Belize may be significant (reciprocally monophyletic at mtCOI and divergent at mt16S and other nuclear loci in this paper), there may be little more to do in describing this disjunction - perhaps an incipient speciation - if few populations exist in between these areas. The divergence between northern and southern populations of *C. southwardorum* is also important and perhaps maintained by the lack of available habitat across southwestern Mexico and northern Central America [35], but given the morphological similarity and broad geographic overlap with other species [7] in the Tropical Eastern Pacific, a phylogeographic survey of this species would probably require significant additional sampling of congeners, but would perhaps help identify the microhabitat that is more predictive of the presence of *C. southwardorum* [36].

An important element of phylogenetic analysis in a widely distributed marine genus such as *Chthamalus* is the ability to expand on the traditional sequence markers that have been used for biogeographic or phylogenetic study. With two sets of trans-Isthmian comparisons to make for all loci considered here, we are able to identify likely evolutionary rates for at least two novel markers that may be useful in subsequent phylogeographic and phylogenetic analysis (Table 1). Outstanding issues that remain to be explored include a more detailed phylogeny incorporating the eastern hemisphere taxa, particularly the “*malayensis* group” (for which additional specimens were available but difficult to curate with respect to recognized intraspecific variation; COI data from *C. malayensis* in this study correspond with the ‘South China Sea’ clade from Tsang et al. [24], but other specimens generated unrecognizable sequence diversity). Pope [15] and Southward and Newman [17] noted that additional investigation is necessary for both the *malayensis* and *challengeri* subgroups, and inclusion of only 2 taxa per group in this study is sufficient only to show that they are likely to be monophyletic groups. However it is also clear that for taxa that have radiated as recently as the genus *Chthamalus*, the search for adequate phylogenetic information is still incomplete. Comparison of genomic data sets from related taxa may improve our ability to identify gene regions with sufficient variability and consistency for full resolution of this phylogenetic problem [37].

Overall, the results of our study point to a pattern that suggests rapid radiation in this genus, particularly along the American coasts, in the past few million years. Further analysis of the TEP species of *Chthamalus* – particularly with regard to the separation of *C. southwardorum* clades across the Central American Faunal Gap [38] – will improve our understanding of the association between species radiation in this group and specific climatic and oceanographic events that may have affected the paleodistributions and interactions of these species [39]. Although little is known about the microhabitat requirements for codistributed species of *Chthamalus* [7], [36], [40], there is an intriguing frequency of closely related species in sympatry in the TEP (Hellberg 1998), which suggests that simultaneous evaluation of speciation and species interactions in this group will be fruitful. We have also recovered, using advanced phylogenetic and deep coalescent approaches, a relationship between the genera *Chthamalus* and *Microeuraphia* that not only suggests taxonomic revision is necessary, but a re-evaluation of our understanding of the morphological evolution of this family of barnacles [18]. The barnacles in the genus *Chthamalus* are small and relatively nondescript, but these high intertidal species have contributed to seminal work in ecology and biogeography, and a complete understanding of the evolutionary history of this group enables a more thorough understanding of the causes and consequences of macroevolutionary patterns in barnacles globally [41].

## Materials and Methods

### Collection and Curation of Specimens

Table 2 lists all sites from which specimens were obtained under permit, along with sample sizes, identification, and GenBank accession information. From each individual, the musculature connecting soma to opercular plates was dissected away, leaving key morphological features (e.g., cirri, trophi) for taxonomic verification. Remaining taxonomically relevant tissues are preserved in 95% ethanol for further examination. Nucleic acids were isolated from musculature using the PureGene protocol (Gentra Systems), with template DNA quantified on a Nanodrop spectrophotometer.

**Table 2:** Collection information for phylogenetic study of *Chthamalus* and related species.

Species	Population(s)	Clade Support BS – PP	mtCOI
*Semibalanus balanoides*	Connecticut, USA (41.17°, −73.21°)	100 – 100	FJ845815-819
*Amphibalanus eburneus*	Georgia, USA (31.48°, −81.28°)	100 – 100	FJ845840-844
*Notochthamalus scabrosus*	Chile (−33.52°, −71.54°)	100 – 100	FJ845820-827
*Microeuraphia eastropacensis*	Panama (8.96°, −79.51°)	100 – 100	FJ845851-862
*M. rhizophorae*	Panama (9.55°, −79.65°), Brazil (−23.15°, −44.75°)	100 – 100	FJ845863-866
*Chthamalus proteus* a	Caribbean Panama (see legend)	100 – 100	FJ858021-040
*C. angustitergum*	Caribbean Panama (see legend)	100 – 100	FJ858041-FJ858059
*C. angustitergum* Belize	Belize (16.81, −88.10)	100 – 100	FJ845832-839
*C. bisinuatus*	Brazil (−23.05, −43.25)	100 – 100	FJ845845-850
*C. malayensis* b	Singapore (1.12°, 103.80°)	100 – 100	FJ848828-831
*C. dentatus* c	Cameroon (3.25°, 9.75°)	100 – 100	FJ858081-088
*C. montagui*	Senegal (14.03°, −17.35°)	100 – 100	FJ858060-067
*C. challengeri*	Japan (42.5°, 144.5°)	100 – 100	FJ858068-076
*C. dalli*	Oregon, USA (42.80°, −124.3°)	100 – 100	AY795282-480^d^
*C. fragilis*	Florida, USA (42.15°, −124.3°)	100 – 100	AF234807-813^e^
*C. southwardorum* A^f^	Tropical Eastern Pacific (see legend)	95 – 100 (*C. s.* A+B 99 – 100)	FJ858001-020
*C. southwardorum* B	Tropical Eastern Pacific (see legend)	94 – 100 (*C. s.* A+B 99 – 100)	FJ857992-FJ858000
*C. hedgecocki* f	Tropical Eastern Pacific (see legend)	63 – 64 (*C.h.*+*C.p.* 100 – 100)	FJ857983-991
*C. panamensis* f	Tropical Eastern Pacific (see legend)	73–87 (*C.h.*+*C.p.* 100 – 100)	FJ857949-982
*C. anisopoma*	Mexico (28.9°, −113.5°)	100 – 100	AF234816-819^e^
*C. fissus*	California, USA (32.83°, −117.35°)	100 – 100	AF234463–527^e^
*C. neglectus*	Hong Kong (22.25°, 114.25°)	100 – 100	FJ858077-080
*C. stellatus*	England (50.33°, −4.12°)	100 – 100	EU699239-250^g^

All taxa were morphologically validated by authors and/or BLAST match to extant sequence data for taxon, and tissues are curated for future analysis, unless marked otherwise. Some species were collected at multiple locations, but are still represented by a single mtCOI clade (unless noted otherwise); bootstrap (BS) and posterior probability (PP) support indices for mtCOI taxon clades are given, followed by Genbank accession numbers for each species at mtCOI. Genbank accessions for the remaining loci are provided as alignments: mt16S FJ862065-306; nLTRS FJ862503-665; nNAKAS FJ862666-774; nEF1 FJ862307-FJ862502. Combined collection locations include Caribbean Panama (sites at 9.33°, −82.25°; 9.55°, −79.65°; and 9.52°, −79.55°) and Tropical Eastern Pacific (8.96°, −79.51°; 9.95°, −84.87°; 19.28°, −104.78°; 19.58°, −105.12°; 20.77°, −105.52°; 23.15°, −106.20°; 24.25°, −110.00°; and 28.77°, −111.88°).

a *C. proteus* analyzed in this study BLAST to clade A of Zardus & Hadfield [43].

b collected and validated Yixiong Cai; corresponds to Indo-Malay clade of Tsang et al. [24].

c validated by A. J. Southward.

d from [36].

e from [21].

f species validated through clade identity with type specimen sequences in [7]. For more information on distribution of individual species see [7] and Meyers, Pankey & Wares (in prep).

g from Shemesh et al. in press.

After coarse morphological identification (cirri I/II, mandible), individual specimens were ‘barcoded’ using universal [42] mitochondrial cytochrome oxidase I (mtCOI) primers for comparison with previous taxonomic and phylogenetic analyses of this genus (e.g., [7], [21], [22], [43]). A sample of individuals from each mtCOI clade (see Table 2) identified as having 100% bootstrap support (neighbor-joining algorthim in GENEIOUS 2.5.4 and/or PAUP*4.0b10, Swofford [44]), 100% consensus support, or 100% posterior Bayesian probability (MrBayes 3.1, [45]) was then sequenced at additional loci. These barcoded OTUs – that in almost every case match their identity based on morphology and sample location – are used independently of subsequent phylogenetic analysis, because studies [21], [22] have illustrated the poor performance of mtCOI for phylogenetic analysis of this group.

### Sequence Collection and Verification

A sample of individuals from each mtCOI clade identified in Table 2 was then sequenced for 5 additional gene regions: mitochondrial 16S rDNA (mt16S F: 5′-CTGTGCTAAGGTAGCATAATCA; R: 5′-AGAAGATAGAAACCAACCTG), nuclear lysidyl aminoacyl transfer RNA synthetase (nLTRS;[46]; F: 5′-CGAATGGATGACACGACGTA; R: 5′-GGATGGGTTCATTTTCAAGG), nuclear eukaryotic elongation factor 1α (nEF1 F: 5′-CAGACGCAGGGGCTTGTC; R: 5′-GCCACAGGGATTTCATCAAG), nuclear r18S [47], and nuclear Na-K-ATPase (nNAKA F: 5′-GTGGTTCGACAACCAGATCA; R: 5′-GGGATCTCGCAGACCTTCTT). These loci were chosen based on previous phylogenetic performance in closely related taxa (16S, 18S, and nEF1; [47]) or were developed from previously published regions that harbor high variability in crustaceans (LTRS, [46], NAKAS, [48]). The newly-developed loci represent non-coding intron data (LTRS) and coding sequence (NAKAS) that harbors substitutions consistent with neutral evolution in interspecific comparisons (McDonald-Kreitman test, p>0.05, Wares, Zakas, and Pankey unpublished results). DNA from 3–10 specimens per species (per location for suspected cryptic species) was analyzed.

PCRs were carried out in 25 µl volumes consisting of 0.5 µM each primer, 0.8 mM total dNTPs, 2 mM MgCl_2_, and 1 U Taq polymerase (Promega). Annealing temperatures for each locuswere as follows: 16S, 50°; COI, 48°; EF1, 52°; LTRS, 47°; NAKA, 46°; r18S, 50°. PCR products were prepared for sequencing using Exonuclease I and Antarctic Phosphotase (NEB). Sequencing reactions were carried out in 10 µl volumes with 80 ng of prepared template, 0.6 µM primer, 0.6 µl BigDye Terminator (Applied Biosystems) and 3.4 µl Better Buffer (The Gel Company). Sequences were cleaned with 4 volumes 75% isopropanol, suspended in Hi-Di formamide (Applied) and run on an ABI 3730.

For each locus, sequence data were edited using CodonCode Aligner v.2.06. All nucleotide data were scored for quality using PHRED [49], with sites having quality <15 declared ambiguous (N). Sequences included for analysis were end-clipped so that only bases with quality scores of 15 and higher were retained. Sequences were aligned using Aligner's built-in ‘end-to-end’ algorithm (with mismatch and gap penalties of −2 and additional gap penalty of −3; other alignment parameters were evaluated with little change in alignment quality), examined and edited for likely artifacts caused by poly-N repeats and other apparent insertions, and disassembled/realigned. In all data sets, gapped characters are removed from analysis. Each data set was then examined for phylogenetic signal using DAMBE [50] and the method for inferring substitution saturation developed by Xia and colleagues [51].

### Phylogenetic Analysis

Each sequence data set was first subjected to maximum parsimony (MP) and uncorrected neighbor-joining (NJ) analysis in PAUP*4.0b10 [44] with 1000 nonparametric bootstrap replicates, using tree-bisection-reconnection rearrangements and a heuristic search, holding the maximum number of equal trees at 100. These preliminary analyses evaluated two components necessary for subsequent species-level analysis. First, we identified sequences that do not appear to consistently group with apparent barcoded conspecifics; these sequences may represent curation errors, artifacts, or chimeric sequences generated naturally. Such sequences were excluded from subsequent analysis (see Results). Additionally, minimum and mean bootstrap values were identified for each locus. There is evidence that these measures are indicators of the likelihood that a data set can recover the true phylogeny [37]. The phylogenetic signal at each locus was evaluated in pairwise comparisons with each other locus using a partition homogeneity test [52] implemented in PAUP*. Each comparison involved 1000 permutations and was repeated with and without constant characters. A concatenated data set (generated with the fused matrix export option in Mesquite and using only individuals for which at least 2 loci were fully sequenced, with missing information coded distinctly from gapped characters) was evaluated for similar performance.

The best-fit model of molecular evolution was assessed for each locus using Modeltest 3.7 [53], using likelihood-ratio tests to choose the simplest model with a significantly improved fit to the data. For subsequent Bayesian analysis, we used MrModeltest [54] instead; similar models were generated for each locus by both model estimation procedures. These models are used independently for each gene region for all subsequent distance, likelihood, or Bayesian phylogenetic analyses discussed below. Distance, parsimony, and likelihood analysis of each data set was performed in PAUP*4.0b10 with random addition, tree-bisection-reconnection branch swapping, and heuristic search methods. For Bayesian analysis, each locus was analyzed separately using MrBayes, with model parameters as chosen above, 4 heated MCMC chains and 2 independent runs in each analysis. Bayesian analysis involved a minimum of 5×10^6^ MCMC generations with sample frequency *f* = 1000; if the standard error between independent runs was not less than 0.05 at this point, additional sets of 1×10^6^ generations were run until this standard was reached. Unlinked concatenated gene analysis on a reduced data set (n = 3+ individuals per species) was analyzed in the same way, as in [29]. The first 25% of generations were discarded as burnin, an approach verified through graphical analysis of the stabilization of likelihood values.

### Bayesian Estimate of Species Tree

Subsequent to traditional phylogenetic analyses, a multi-locus approach that allowed for distinct genealogical histories within the species tree (and used the variation among gene trees as information regarding the interaction of ancestral effective population size and time between nodes on ability to resolve those nodes) was attempted (see [55] for review). This approach uses mb-best v. 2.0 [30], a modification of MrBayes. As before, this Bayesian analysis used unlinked models of molecular evolution for each locus. Four initial attempts were used to generate the best-performing set of prior distributions on per-gene theta, with each run requiring more than 30 days of computational time on 2.0 GHz dual-processor Macintosh computers. Runs utilized 2×10^7^ generations with four MCMC chains and a sample frequency *f* = 1000 trees. We chose an inverse gamma with parameters [2,1] for theta in our final analyses, with a default uniform prior [0.5,1.5] on gene mutation rate.

### Species Radiation Timing

To evaluate the rate of molecular evolution at each locus, standard likelihood ratio tests were performed on individual loci as in [21], utilizing the best-fit model and the same model constrained by clock assumptions. Following these tests, trans-Isthmian comparisons were made of the geminate pair of *Microeuraphia* species, and for all pairs of trans-Isthmian *Chthamalus* species; the latter arrangement is based on speciation that has taken place in the Caribbean (*C. proteus/C.fragilis*) and Pacific (*C. fissus*, *C. anisopoma*, and the TEP species of [7]) subsequent to closure of the Panamanian Isthmus. Hence, the net nucleotide divergence for all species pairs was calculated in DNAsp v.4.20 [56] and averaged for the *Chthamalus* comparison, with standard deviation estimated and summarized the same way. Rate estimates are based on the range of closure/isolation dates from 3.0–3.5 mya [57].

The final concatenated topology was evaluated using analysis of speciation and extinction in the BiSSE character-independent model [58]. This analysis generates relative rates of speciation and extinction through time, and calculates the likelihood that one rate is higher than the other for a given phylogeny with branch lengths. This analysis was repeated using the character-associated BiSSE model to examine different levels of diversification rates between the mostly American ‘fissus group’ and remaining taxa on the phylogeny.
